# The Rate and Effects of Spontaneous Mutation on Fitness Traits in the Social Amoeba, *Dictyostelium discoideum*

**DOI:** 10.1534/g3.113.005934

**Published:** 2013-07-01

**Authors:** David W. Hall, Sara Fox, Jennie J. Kuzdzal-Fick, Joan E. Strassmann, David C. Queller

**Affiliations:** * Department of Genetics, University of Georgia, Athens, Georgia 30602; † Department of Ecology and Evolutionary Biology, Rice University, Houston, Texas 77005; § Department of Systems Biology, The University of Texas MD Anderson Cancer Center, Houston, Texas 77054; ‡ Department of Biology, Washington University in St. Louis, St. Louis, Missouri 63130

**Keywords:** mutation accumulation, mutation rate, fitness components, *Dictyostelium*

## Abstract

We performed a mutation accumulation (MA) experiment in the social amoeba *Dictyostelium discoideum* to estimate the rate and distribution of effects of spontaneous mutations affecting eight putative fitness traits. We found that the per-generation mutation rate for most fitness components is 0.0019 mutations per haploid genome per generation or larger. This rate is an order of magnitude higher than estimates for fitness components in the unicellular eukaryote *Saccharomyces cerevisiae*, even though the base-pair substitution rate is two orders of magnitude lower. The high rate of fitness-altering mutations observed in this species may be partially explained by a large mutational target relative to *S. cerevisiae*. Fitness-altering mutations also may occur primarily at simple sequence repeats, which are common throughout the genome, including in coding regions, and may represent a target that is particularly likely to give fitness effects upon mutation. The majority of mutations had deleterious effects on fitness, but there was evidence for a substantial fraction, up to 40%, being beneficial for some of the putative fitness traits. Competitive ability within the multicellular slug appears to be under weak directional selection, perhaps reflecting the fact that slugs are sometimes, but not often, comprised of multiple clones in nature. Evidence for pleiotropy among fitness components across MA lines was absent, suggesting that mutations tend to act on single fitness components.

Spontaneous mutations are the ultimate source of all genetic variation, making them of paramount importance at every level in biology, from genetic disease to evolution. Of particular interest for understanding evolution are those mutations that affect fitness and are therefore visible to natural selection (reviewed in Lynch *et al.* 1999). Many models predict particular values of mutational parameters if they are to explain various evolutionary patterns. Kondrashov’s deterministic mutation hypothesis for the maintenance of sexual reproduction requires that the genome-wide rate of deleterious mutations is greater than 1 (Kondrashov 1988). The nearly neutral theory of molecular evolution (Ohta 1992) requires that a large class of mutations have small selection coefficients such that they are effectively neutral in small but not large populations. The manner in which genetic load is purged depends on whether inbreeding depression is due to large or small effect deleterious mutations (Hedrick 1994).

In estimating parameters of spontaneous mutations affecting fitness, researchers often use mutation accumulation (MA) experiments (Garcia-Dorado *et al.* 1998; Keightley 1994; Mukai 1964; Simmons and Crow 1977). MA experiments maintain lines under extremely low effective population size to minimize the effects of selection, reducing its efficacy so that mutations of small-to-moderate effect accumulate at a rate approximately equal to their occurrence (Lynch *et al.* 1999). After many generations of accumulation, the fitness of each MA line is measured. The expectation is that MA lines will show reduced fitness relative to the ancestor because if an organism is currently well adapted to its environment, then mutations are most likely to reduce fitness (Fisher 1930). The reduction in the mean and increase in the variance of fitness among lines are then used to estimate the parameters of mutations affecting fitness (Bateman 1959; Keightley 1994; Keightley and Ohnishi 1998; Mukai 1964).

MA experiments have been performed using a variety of species, from microbes to flies, but not very many studies have been undertaken (Table 1). The data that have been collected suggest that the genome-wide rate of mutations affecting fitness (*U*) is larger in multicellular eukaryotes than in microbial eukaryotes. However this conclusion rests heavily on the low mutation rate of the yeast *Saccharomyces cerevisiae* (*Tetrahymena thermophila* has a much higher rate, but this is for its somatic macronucleus rather than the germline rate reported for all other species). Determining the robustness and the cause of this pattern is an important question. There are several factors that may contribute to the differences in *U* among species, including differences in the number of cell divisions per generation (Drost and Lee 1995), the molecular mutation rate, the spectrum of mutations, the number of genes, and the proportion of the genome that is coding *vs.* noncoding (summarized in Lynch 2006). However, with data on only a handful of species, and overlap among hypotheses, distinguishing among them is difficult. Data from additional taxa are needed both to determine whether the pattern is robust and to elucidate its cause.

**Table 1:** Some MA estimates of haploid mutation rates per generation, *U*, and their average effects, E(*a*), for mutations that affect fitness

Taxon	Fitness Component	*U*	E(*a*)	Reference
*D. melanogaster*	Viability	0.35	0.027	Mukai 1964
	Viability	0.47	0.023	Mukai *et al.* 1972
	Viability	0.14	0.03	Ohnishi 1977
	Viability	0.02	0.1	Garcia-Dorado *et al.* 1998
	Viability	0.052	0.11	Fry *et al.* 1999
	Viability	0.29	0.02	Charlesworth *et al.* 2004
*A. thaliana*	LRS	0.05	0.23	Schultz *et al.* 1999
	Fruit number	0.06	0.06*^a^*	Shaw *et al.* 2002
*C. elegans*	r	0.0035	0.1	Keightley and Caballero 1997
	*r*	0.008	0.2	Vassilieva and Lynch 1999
	*r*	0.024	0.131	Estes *et al.* 2004
	*r*	0.0033	0.182	Baer *et al.* 2005
	*r*	0.0042	0.126	Baer *et al.* 2005
	Productivity	0.018	0.369	Estes *et al.* 2004
	Survival	0.003	0.390	Estes *et al.* 2004
*C. briggsae*	r	0.037	0.051	Baer *et al.* 2005
	*r*	0.013	0.099	Baer *et al.* 2005
*O. myriophila*	*r*	0.0028	0.219	Baer *et al.* 2005
*T. thermophila*	*r*	0.033	0.16	Brito *et al.* 2010
*S. cerevisiae*	MGR	0.00006	0 0.061*^a^*	Joseph and Hall 2004
	MGR	0.00014	0 0.073*^a^*	Hall *et al.* 2008
	SE	0.00019	0.70	Hall and Joseph 2010
	*r*	0.00013	0.79 *^b^*	Hall and Joseph 2010
	*r*	0.00055	0.086*^b^*	Wloch *et al.* 2001
	*r*	0.000048	0.217*^a^*	Zeyl and DeVisser 2001
	*r*	–	0–0.049*^a^**^c^*	Zeyl and DeVisser 2001
*E. coli*	*r*	0.00017	0.012	Kibota and Lynch 1996

The effect of mutations is measured in homozygotes, except where noted. Table modified from Bataillon (2000). MA, mutation accumulation; LRS, lifetime reproductive success; *r*, growth rate; MGR, maximum growth rate; SE, sporulation efficiency.

a Mean effect in heterozygotes.

b Mean effect in haploids.

c Data from a mutator line.

Protists are extremely diverse and thus represent an excellent group in which to address the causes of mutation rate variation. Of particular interest is that some protists, such as *Dictyostelium discoideum*, exhibit both unicellular and multicellular stages in their life cycle. If multicellularity results in increased mutation rates, we might expect to see mutation rates in *D. discoideum* that are intermediate between unicellular and multicellular species. Nucleotide diversity in *D. discoideum* (Evans *et al.* 1988; Flowers *et al.* 2010; Francis and Eisenberg 1993) and in *S. cerevisiae* (*e.g.*, Winzeler *et al.* 1998) are similar, indicating that the product of the molecular mutation rate (*μ*) and the effective population size (*N*_e_) is similar in the two species. The base-pair substitution rate is substantially lower in *D. discoideum* than in *S. cerevisiae*, perhaps by two orders of magnitude (Saxer *et al.* 2012), which suggests that its effective population size is substantially larger. One question is whether this low base pair substitution rate is reflected in a low rate of fitness-altering mutations. One other protist for which there is an estimate, *Paramecium tetrauelia* has a similarly low base pair substitution rate per cell division (Sung *et al.* 2012).

*D. discoideum* is a eukaryotic, haploid, single-celled amoeba that lives in the soil, preys on bacteria, and divides mitotically. When food becomes scarce, *D. discoideum* amoebae send out a chemical signal, cAMP, that causes all cells in the vicinity to join together in a multicellular structure termed a slug that is able to migrate toward heat and light (Kessin 2001; Raper and Rahn 1984). When the slug finds a suitable location, it undergoes differentiation into a fruiting body (FB) in which roughly 20% of the cells die to form a vacuolated stalk that holds up the remaining 80% of cells in a ball of reproductive spores. The stalk functions to raise the reproductive spores away from the forest floor, facilitating their dispersal by passing organisms (Bonner 1982; Huss 1989). Once spores have been dispersed, they germinate to produce new amoebas. They also have a meiotic sexual stage that we are not studying here.

Given its rather complex life cycle, *D. discoideum* has many candidate fitness components. We measured eight of them. They included growth rate (*i.e.*, doubling time) during the single-cell stage, measured in two conditions, the distance the multicellular slug is able to move across a nutrient-free agar plate, the total number of spores produced, the number of FBs, and the number of spores per FB. We also measured germination ability as a fitness component for the spore stage.

The final trait measured requires some explanation. Multicellular development in *Dictyostelium* is different from most other organisms because aggregation can bring together different clones. These genetically distinct clones may have conflicting interests with regard to spore production (Strassmann and Queller 2011a; Strassmann *et al.* 2000). For example, clones may have evolved exploitative mechanisms in an attempt to increase the odds that they will end up as spores rather than in the reproductive dead-end of the stalk (Foster *et al.* 2002; Strassmann 2000). Genetically different clones can mix and form chimeras in nature (Fortunato *et al.* 2003b; Strassmann *et al.* 2000), despite some segregation (Benabentos *et al.* 2009; Ostrowski *et al.* 2008). However, we do not know exactly how important competition between clones is in the wild, as Gilbert *et al.* (2007) reported that most, though not all, FBs collected in the field from dung were clonal. We thus measured this competitive ability to see if it behaved like a fitness component under MA.

In the MA experiment we independently passaged 90 initially identical lines for ~1000 cell generations including 70 single-cell bottlenecks. The changes in the traits were then used to estimate parameters of mutation for each fitness component. We were also able to determine whether there was evidence for pleiotropy among accumulated mutations.

## Materials and Methods

### Mutation accumulation

The MA experiment was previously described in a study of the mutation rate at microsatellite loci (Mcconnell *et al.* 2007). We initiated the MA experiment from a single cell of the laboratory-generated strain AX4 (Knecht *et al.* 1986) of *Dictyostelium discoideum*, provided by Gad Shaulsky at Baylor College of Medicine. AX4 is an axenic strain that, in addition to feeding on bacteria, can be grown in standard HL5 liquid medium (Watts and Ashworth 1970) without a bacterial food source. A single isolate served as the ancestral clone for all MA and control lines. We used 90 MA lines and 10 control lines. A sample of the original clone was starved to stimulate FB formation, and then spores were stored at −80° using standard methodology (Fey *et al.* 2007). We passaged all lines at 22° on standard Petri dishes (100 × 15 mm) containing SM agar (Sussman 1966) and *Klebsiella pneumoniae*, which was used as a bacterial food source for the amoebae.

Each MA line was put through a single-cell bottleneck every 48 hr for a total of 70 bottlenecks. There were 14.2 cell generations per transfer (Mcconnell *et al.* 2007) for a total of 994 cell generations. Transfers occurred before fruiting, so no fruiting occurred in the MA lines and no selection occurred on multicellular traits. Every 10 transfers, each line was transferred onto two plates. One plate was used to continue the MA, whereas the other was used to generate spores for long-term storage by allowing amoebae to produce FBs, from which spores were collected and stored at –80° (Fey *et al.* 2007). The control lines were handled in the same manner as the MA lines, except that a higher minimum population size was maintained. At each transfer many colonies were transferred by sweeping a loop across the plate, including regions where colonies were so dense as to be indistinguishable. The effective population size was ~7.5 in MA lines and substantially larger, but unknown, in the control lines. Higher effective population size reduces the probability that deleterious mutations will fix, allowing us to determine the extent to which selection is occurring on each fitness component in our MA environment. If control lines are more similar to the ancestor than the MA lines, it is evidence that selection prevented them from accumulating deleterious mutations. However, aside from plate growth, none of the traits we scored were expressed during the passage of the MA lines, so the only selection that could occur on these traits would be indirect selection via correlated traits.

At the end of the MA phase of the experiment, we scored MA lines, control lines, and the ancestor for eight putative fitness components, described below in *Fitness components*. For each, the abbreviation used for that fitness component is given. There were too many lines to score a fitness component on all strains in a single block. To control for possible discrepancies in laboratory conditions across blocks, the ancestor was always assayed in parallel with each set of lines scored.

### Fitness components

To measure the eight fitness components, MA lines were first recovered from the freezer by plating spores at low density on SM plates with *K. pneumoniae* as food and growing them for 48 hr. For each of the fitness components except competitive ability, the relative fitness for each MA line was obtained by dividing its fitness estimate by the average of the ancestor. For all of the fitness components, relative fitness was used to estimate parameters of mutation.

#### Growth on SM agar plates containing a lawn of K. pneumoniae (plate growth):

For each line, a randomly chosen plaque was transferred to a SM plate with *K. pneumonia*, grown for 48 hr, and cells from a single, random plaque were then transferred to a new plate. After another 48 hr, five random plaques were each suspended in 50 μL of KK2, and the average number of cells in a plaque was estimated with the use of a hemocytometer. Cell number was then converted to growth rate, or the number of doublings that occurred in the 48 hr of growth, which is equal to Log(cell number)/Log(2), assuming each plaque was founded by a single amoeba.

#### Growth in HL5 medium (liquid growth):

Our ancestral strain is able to grow in axenic medium, which lacks a bacterial food source. Growth in axenic medium is stressful compared with growth on bacteria, and doubling times are often two to three times slower for those strains that are able to grow in axenic medium (Fey *et al.* 2007). We scored growth in this environment because stressful environments can uncover deleterious fitness effects of new mutations (Szafraniec *et al.* 2001). Actively growing cells in a plaque were transferred into 10 mL of HL5 in a Petri dish. After 5 d of growth, the entire volume was transferred to a flask and brought to a total volume of 25 mL HL5. Cells were then grown with shaking at 22° for ~24 hr. These steps allowed the strains to recover from freezing and insured that they were growing as rapidly as possible. Next, an aliquot was removed and diluted to a density of 10^6^ cells in 50 mL of HL5 and returned to the shaker. The cell density of the culture after an additional 24 hr of growth was then used to calculate the number of doublings during the final 24 hr.

#### Total distance traveled by a slug (slug distance):

We first added 5 × 10^6^ cells to a “starting line” on one side of a plain agar (no additives) plate. The starting line consisted of 50 μL of a slurry of *K. pneumoniae* pipetted onto on a 2-cm strip. Each plate was wrapped in aluminum foil with a pin-hole at the opposite end from the starting line to allow light to enter. Cells rapidly exhausted the food supply and formed slugs that migrated toward the light. Plain agar medium was used to increase the average distance moved (Slifkin and Bonner 1952). All plates were housed in a lighted Percival Intellus Environmental Controller incubator set at 22° with pin-hole openings facing away from the door to minimize differences in light intensities. We measured the average distance of FBs from the starting line after 5 and 13 d, but only the 5-d data were used, because dessication made counts difficult by 13 d.

#### Total number of FBs:

We counted the total number of FB on the plates of the slug distance experiment.

#### Total number of spores per FB:

With an insect pin, we carefully lifted 10 FB from the slug distance plates, suspended them in buffer, and then counted the number of spores with a hemacytometer.

#### Total number of spores:

We picked up all the remaining FB on the slug distance plates, resuspended them in buffer, and then counted total spores with a hemacytometer.

#### Spore germination rate (spore germination):

To acclimate each line, spores were plated at low density on SM plates and left long enough to form FB. Spores from these FBs were then replated to produce new FBs. After the second round of fruiting, spores were collected and diluted to a density of 10^3^ spores/mL. Spores were then plated onto two SM plates, 50 spores per plate spread evenly with *K. pneumoniae* as a food source. Each day plates were checked for plaques, indicating spore germination. Germination usually occurred in the first 2 d. We continued to score each plate until no new plaques appeared over three consecutive days. Across all strains, we never observed germination after 5 d postplating. Germination rate of each line was scored twice for a total of 200 spores.

#### Ability to compete against the ancestor for presence in spores (competitive ability):

To test the competitive ability of MA and control lines in the multicellular stage, pairwise mixtures of each line with the AX4 ancestor were competed. To summarize, cells for the line and the ancestor were collected, labeled (one or the other strain), mixed, and starved to stimulate slug and FB formation, and then spores in the FB were counted to measure the relative contribution of the two strains.

Specifically, strains were grown for 40 hr from an initial density of ~3 × 10^5^ cells/plate on a *K. pneumoniae* lawn on SM plates. This cell density is well below the density required to initiate cellular differentiation. All cells on the plate, which were not starved and still dividing, were then collected into a 50-mL Falcon tube and washed three times by centrifugation and resuspended in KK2 solution (16.5 mM KH_2_PO_4_, 3.8 mM K_2_HPO_4_) (Fey *et al.* 2007). After the third wash, the density of the cell suspension was measured using a hemocytometer and diluted to a final concentration of 10^7^ cells/mL.

A sample of each strain was then labeled with Cell Tracker (Molecular Probes, Inc.), a fluorescent dye that binds inside the cell membrane and remains bound through sporulation (Mehdiabadi *et al.* 2006). In addition to equal mixes of the two competing strains, one labeled and one unlabeled (in both directions), several control treatments were performed. Equal mixes of labeled and unlabeled cells of each strain alone, and pure cultures of labeled or unlabeled cells of each strain were made. Cells were visually inspected under a Nikon E1000 fluorescence microscope to confirm high initial levels of labeling in each experiment.

Cells from each treatment were placed into starvation medium to stimulate FB formation (Fey *et al.* 2007). For each treatment ~2 × 10^6^ cells/mL (1 × 10^6^ of each type in mixes) were placed on a nitrocellulose filter on top of a filter pad soaked with 1 mL of PDF medium (20.1 mM KCL, 5.3 mM MgCl_2_•6H_2_O, 9.2 mM K_2_HPO_4_, 13.2 mM KH_2_PO_4_, 0.5 g/L streptomycin sulfate, pH to 6.4), in a Petri dish. Dishes were kept in the dark at 22° and amoebas were allowed to starve and produce FBs. Once fruiting was complete, which occurred in fewer than 2 d, all FBs from each plate were collected in 200 mL of KK2 + 10 mM ethylenediaminetetraacetic acid. The density of the spores was determined using a hemocytometer.

For each competition treatment, the number of labeled cells (before starvation) of a total of at least 500, and the number of labeled spores (after the formation of FBs) of at least 500, were counted to estimate the relative contribution of the labeled strain to spore production. The efficiency of label retention also was estimated in the single-strain, fully labeled treatment, by scoring the number of 250 spores that were not labeled. In the control treatments for the competition experiments, 99.2% of spores retained the Cell Tracker dye on average (SD 0.31, n = 182). For 20 of the 90 MA lines, the competition treatments were repeated and the two replicates averaged.

To measure the competitive ability of a line, *C_line_*, we divided the ratio of line (*S_line_*) to ancestor (*S_anc_*) spores by the ratio of line (*N_line_*) to ancestor (*N_anc_*) cells in the initial mixture

Cline=SlineSancNlineNanc. (1)

Competitive ability is an index of cheating. Values less than 1 indicate a poor competitor/cheater relative to the ancestor, and values greater than 1 indicate a good competitor/cheater. The competitive ability for a line is the average across the two reciprocal mixes. Other competition indices also were tested, but no difference in estimates of mutation parameters was found among them (data not shown).

### General statistical analysis

Several data analyses were performed. First, distributions were generated for each putative fitness component. The MA line distribution consisted of 90 points representing the relative fitness for each MA line, averaged across replicates when present. The control line distribution consisted of 10 points and represented the fitness of the 10 control lines, similarly averaged across replicates. The ancestor distribution consisted of the relative fitness of each replicate of the ancestor strain for all measures except competitive ability. For competitive ability, every MA line measure is the average of two, reciprocally labeled replicates and so we used random pairs of ancestor replicates (50% labeled ancestor competed against 50% unlabeled ancestor) for this distribution. The number of ancestor replicates measured varied across fitness components and so the size of the sample in each ancestor distribution varies (Table 2).

**Table 2:** Comparison of means and variances of the ancestor, control, and MA line distributions

	Ancestor		Control (n = 10)		MA Lines (n = 90)	
Fitness Component	Mean	Var	Mean	Var	Mean	Var
Plate growth	1.000 (*n* = 16)	0.0015	0.991^NS^	0.0011^NS^	1.000^NS/NS^	0.0025^NS/NS^
Liquid growth	1.000 (*n* = 16)	0.0011	0.922^NS^	0.0275*	0.705***^/^**	0.0471***^/^*
Slug distance	1.000 (*n* = 12)	0.0017	0.867***	0.0030^NS^	0.889***^/NS^	0.0245***^/^*
Total FBs	1.000 (*n* = 9)	0.0014	0.902**	0.0016^NS^	0.969 ^NS/^*	0.0112**^/^**
Spores per FB	1.000 (*n* = 12)	0.0017	1.033^NS^	0.0090^NS^	0.919*^/^*	0.0290***^/^*
Total spores	1.000 (*n* = 12)	0.0010	0.930**	0.0021^NS^	0.869***^/^*	0.0080**^/NS^
Spore germination	1.000 (*n* = 17)	0.0000	0.987**	0.0003**	0.519***^/^***	0.0715***^/^***
Competitive ability	0.999 (*n* = 42)	0.0007	1.000^NS^	0.0004^NS^	0.900**^/^**	0.1247***^/^***

Significance of control *vs.* ancestor is given in the superscript of the fourth (mean) and fifth (variance) columns. Significance of MA line *vs.* ancestor (left side of slash) and MA line *vs.* control (right side of slash) are given in superscript of the sixth (mean) and seventh (variance) columns. Significance levels for means are from Wilcoxon nonparametric tests of equality of means, after we corrected for multiple comparisons (Benjamini and Hochberg 2000). Significance levels for variances are from nonparametric Levene tests of equality of variances, after correcting for multiple comparisons. MA, mutation accumulation; Var, variance; FB, fruiting body; NS, not significant. NS *P* > 0.05, *P < 0.05, **P < 0.01, ***P < 0.001.

MA line distributions were generally not normally distributed and so we used nonparametric statistical tests when testing for differences among distributions. To test for differences between blocks for a particular fitness assay, we used the Kruskal-Wallis test on the ancestral strain implemented in R (R 2.4.1 GUI 1.18, 2006). To estimate whether the mean fitness for a particular trait was different between MA lines, control lines and the ancestor replicates, non-parametric Wilcoxon rank sum tests, implemented in JMP (Jmp 2011), were used. To test for equality of variances between MA lines, control lines and the ancestor distributions, a nonparametric version of a Levene’s test was performed (Nordstokke and Zumbo 2010). To do this for a particular fitness component, data were first transformed by subtracting the median value for its respective group (ancestor, control, or MA line), then combining all data from the three groups and converting to ranks. Distributions of the rank scores, in their respective groupings, were then used to perform a Levene’s test in JMP.

To test whether each individual MA line was significantly different from the ancestor, we computed the 95% confidence intervals for the ancestral distributions for each fitness component, assuming normality, and accounting for multiple tests (Benjamini and Hochberg 2000). MA lines falling outside of the interval were deemed to be significantly different from the ancestor for that component.

### Pleiotropy

Some mutations that reduce one fitness component may do so because they are generally deleterious. If a large percentage of mutations are generally deleterious, then lines accumulating such mutations should show reductions in fitness across multiple fitness components, *i.e.*, positive pleiotropy. Another possibility is that mutations have antagonistic effects, such that MA lines may show improvement for one fitness component and reduction for another. To examine pleiotropy, among line, pairwise correlations across fitness components were calculated.

Significant correlations support either pleiotropy or nonindependence of traits. If two traits are not independent, then a positive among-line correlation is not necessarily evidence of pleiotropy. Instead, mutations may affect one underlying trait (no pleiotropy), but because of which traits are measured, two may show a change. For example, if a mutation reduces the number of FBs, it might necessarily also reduce the total number of spores. To gain insight into this issue, we calculated the effective number of traits, *N*_eff_, using the correlations among traits (Wagner *et al.* 2008). The effective number of traits is calculated from the variance in the eigenvalues of the correlation matrix, Var(λ), using

Neff=N−Var(λ), (2)

where *N* is the number of traits in the correlation matrix. We measured eight traits, therefore, if the effective number is 7 or less, that indicates dependence between two or more traits.

We also used simulations in Mathematica (Wolfram Research Inc. 2010) to determine whether there was evidence for pleiotropy. We tested whether our observed data, with possible trait dependencies, matched simulated data with independent trait effects. We did this in two ways. First we counted the number of lines that were significantly different from the ancestor for each trait. Then, we assigned significantly different traits to MA lines at random. For example, two lines are significantly different from the ancestor for plate growth. We thus assigned significant plate growth differences to two MA lines at random in each simulation. We did this for all eight fitness components and then counted how many simulated MA lines differed from the ancestor for 0–8 traits. The numbers we obtained were then compared with the actual number obtained in our experiment with a χ^2^ test, with the simulated data being the expectation under independence. We repeated this for 1000 randomizations and determined how many χ^2^ tests were significant at the 5% level. If there is no pleiotropy, we expect ~95% of simulated data sets to be consistent with the observed data.

Second, we used the number of MA lines that did not differ significantly from the ancestor for each fitness component to obtain a point estimate for the mutation rate for that fitness component. The expected proportion of mutation-free lines is equal to (1 − *μ_i_*)*^G^*, where *μ_i_* is the per-generation mutation rate for fitness component *i*, and *G* is the total number of cell divisions during MA. Each estimated mutation rate was used to parameterize a Poisson distribution for each fitness component. We then used these distributions to simulate the occurrence of fitness-altering mutations in the 90 MA lines. We assumed mutations for each fitness component were independent. After simulating each fitness component, we counted how many lines had 0–8 fitness components that were significantly different from the ancestor. We compared the numbers in each category to the observed using a χ^2^ test, with the simulated data again being the expectation under independence. We repeated this for 1000 randomizations and determined whether observed and simulated data were usually consistent using χ^2^ tests.

### Estimation of parameters of mutation

For each fitness component, log likelihood was used to estimate the proportion of mutations that are beneficial (*P*), the genome-wide rate of fitness-altering mutations (*U*), and the absolute value of the mean fitness effect of mutations [E(*a*)]. Maximum likelihood (ML) estimates were calculated with the use of a program provided by Dr Peter Keightley (Keightley 1994; Keightley and Ohnishi 1998). The likelihood program estimates mutation parameters from the fitness values of the MA lines and the ancestor. The number of mutations accumulated in each MA line is assumed to be Poisson-distributed and the effects of mutations to follow a gamma distribution, reflected around 0 (no effect). A fraction *P* of mutations is assumed to have positive (beneficial) effects. A MA line accumulating a single mutation with a negative effect, *i.e.*, a deleterious mutation, will exhibit relative fitness less than 1.0 and vice versa for a MA line accumulating a single positive effect mutation. The positive and negative parts of the distribution have the same scale parameter *α* and shape parameter *β*. The mean fitness effect, E(*a*), is equal to *β* /*α*. Each data set used in the likelihood analysis consisted of the relative fitness values from the 90 MA lines and between 9 and 42 ancestor fitness measures (Table 2). A search of the parameter space involved first performing the analysis for all 60 combinations of fixed values of *β* (0.1, 0.5, 1, 2, 3, 4, 6, 8, 10, and 50) and *P* (0, 0.1, 0.2, 0.3, 0.4, and 0.5) and then finding the ML values of *α* and *U*. After determining the region of the parameter space in which particular combinations of *β*, *P*, *α*, and *U* showed high likelihoods, we narrowed parameter differences during successive searches to obtain more accurate estimates of the ML values of the parameters and their 95% confidence intervals. In some cases this led to values of *β* or *P* outside of their initial ranges. Additionally, we ran an equal effects model for all values of *P*.

The mutation rate and average effect also were estimated using the Bateman–Mukai approach (Bateman 1959; Mukai 1964). This is a standard approach in MA experiments, and it uses the changes in the mean MA line fitness and the among line variance to generate parameter estimates. The approach does not include the possibility of beneficial mutations. In addition, variance in mutational effects causes the Bateman–Mukai approach to underestimate the genome-wide mutation rate and overestimate the average effect of mutations, because it assumes equal effects (Lynch *et al.* 1999).

## Results

### Statistical preliminaries

Three fitness measures were scored in two (liquid growth and slug distance) or three (plate growth) blocks. Ancestor replicates were also scored in each block, and in no case was there a significant block effect for ancestors: Kruskal-Wallis χ^2^ = 3.44 (df = 2), 2.26 (df = 1), and 3.64 (df = 1), *P* = 0.18, 0.13, and 0.41, respectively, for plate growth, liquid growth, and slug distance. All block data were combined for further analyses.

Tests for outliers among the ancestor replicates for each fitness component revealed one replicate for spore germination in which a measurement was more than 4 SD from the mean (mean germination 95%, SD 19.3%). This replicate was discarded to give 17 replicates used for further analysis (without outlier, mean 99.9%, SD 0.3%).

### Distributions and tests for normality

**Figure 1:**
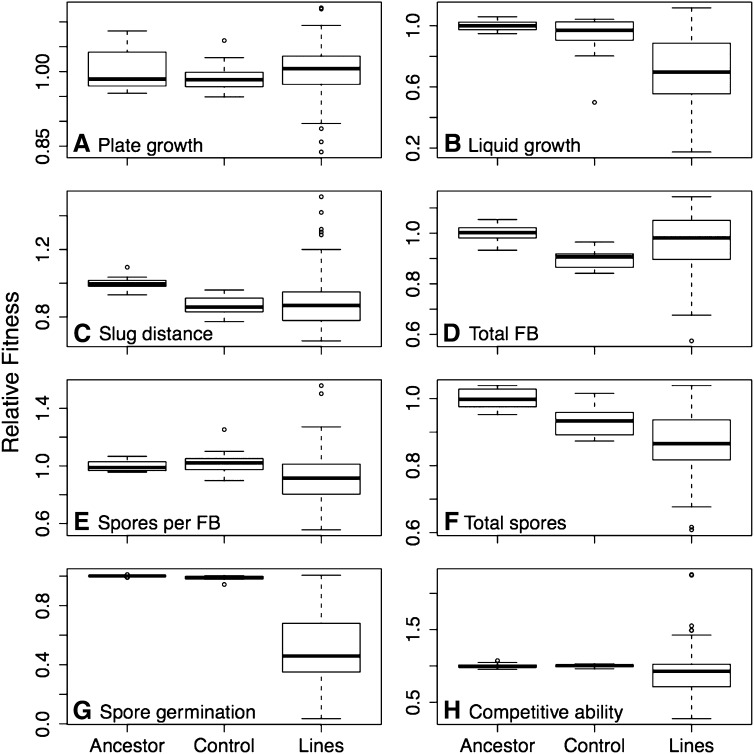
Boxplots of relative fitness distributions for the ancestor, control lines and MA lines for the eight fitness components. Box indicates first and third quartile, horizontal line indicates median. Whiskers extend from the minimum and maximum points lying within 1.5 times the interquartile range below the first and above the third quartiles. Points outside of this range are shown as open circles.

The distributions of relative fitness obtained for all eight putative fitness components are shown as box plots in Figure 1 (histograms are shown in Supporting Information, Figure S1). Ancestor distributions were generally consistent with normality after correcting for multiple comparisons (MC). The one exception was spore germination (spore germination, Shapiro-Wilk W = 0.385, MC corrected *P* < 0.001). The absolute mean of this measure (99.9%) was very close to its maximum possible value (100%): 15 of 17 replicates had 100% germination, and two had 99% germination. With the mean essentially at the maximum possible value for the variable, normality was not expected because values greater than the mean are not possible. Control distributions were also generally consistent with normality. The one exception was doubling time in axenic medium (Liquid growth, Shapiro-Wilk W = 0.723, MC corrected *P* < 0.05), which violated normality due to a single control line showing very slow growth in one of three replicates. MA line distributions were generally not normal. Only two fitness components (liquid growth and total number of spores) had distributions consistent with normality, all others rejected normality: MC corrected *P* < 0.05 for plate growth, *P* < 0.01 for spores per FB, total number of FB and spore germination, and *P* < 0.001 for slug distance and competitive ability.

### Changes in the mean

Accumulation of deleterious mutations is expected to reduce MA line fitness. To examine this prediction, we tested whether the MA lines had a significantly lower mean than the ancestor. Results are shown in Table 2. For six of the eight fitness components, the prediction held and the average MA line fitness was significantly lower than the ancestor. For two components (plate growth and total number of FB), there was no significant change in the mean. The control lines, in which the effective population size was substantially larger than the MA lines, were expected to resemble the MA lines if there was no direct or indirect selection acting on the fitness component during MA. To test for this, we determined whether their means were significantly different from both the ancestor and MA line means (Table 2). The control line mean was significantly lower than the ancestor mean for four of the fitness components (slug distance, total number of FB, total number of spores, and spore germination). Compared instead with the MA line mean, the control line mean was significantly higher for five fitness components (liquid growth, spores per FB, total number of spores, spore germination, and competitive ability) and significantly lower for one fitness component (total number of FB).

### Changes in the variance

Accumulation of deleterious mutations is expected to increase variance among MA lines. To examine this prediction, we tested whether MA lines had a significantly higher variance than the ancestor (Table 2). For seven of the eight fitness components, the prediction held, and the MA line variance was significantly greater than the ancestor. The only exception was growth rate in the experimental environment (plate growth). The control lines were also expected to resemble the MA lines with respect to the variance among lines, if there was no direct or indirect selection acting on the fitness component during MA. To test for this, we determined whether the control line variances were significantly different from ancestor and MA line variances (Table 2). The control line variance was significantly higher than the ancestor for two fitness components (liquid growth and spore germination). The control line variance was significantly lower than the MA line variance for six fitness components (liquid growth, slug distance, total number of FB, spores per FB, spore germination, and competitive ability).

### Number of MA lines with altered fitness

To determine whether a particular MA line is consistent with the ancestor distribution, we calculated the probability that its value would be randomly drawn from a normal distribution with the mean and variance equal to the ancestor’s. Because there are 90 MA lines, we set cut-offs for significance based on a Bonferroni correction (Rice 1989), which implied that a value more than 3.48 SDs from the mean was significant at the 5% level. With this cut-off, the number of MA lines that were different from the ancestor could be determined (Table 3). The range across fitness components was large: only two MA lines differed for plate growth, whereas 85 differed for spore germination. Furthermore, the variation in the percentage of different MA lines that had higher fitness, possibly indicating beneficial mutations, was also large: from 0 (plate growth and spore germination) to 28% (= 17/61 for competitive ability).

**Table 3:** 95% CI bounds assuming normality and corrected for multiple tests, for each fitness component, and the number of lines (of 90) that are significantly below (low) or above (high) the CI

Fitness Component	Lower CI Bound	Upper CI Bound	Low Lines	High Lines
Plate growth	0.867	1.133	2	0
Liquid growth	0.886	1.114	67	1
Slug distance	0.858	1.142	42	6
Total FBs	0.871	1.129	14	3
Spores per FB	0.858	1.142	35	8
Total spores	0.968	1.032	76	1
Spore germination	0.993	1.007	85	0
Competitive ability	0.911	1.089	44	17

CI, confidence interval; FB, fruiting body.

### Pleiotropy

Pairwise correlations are shown in Table 4. Only three of the 28 pairwise correlations are significantly different from zero after correcting for multiple tests. Two of these (total number of FB *vs.* total number of spores and spores per FB *vs.* total number of spores) are not unexpected: mutations that decrease the number of FBs or the number of spores per FB both reduce the total number of spores produced. This finding suggests these three components are not independent. However, the analysis of the effective number of traits using the full correlation matrix indicated that the effective number was ~7.7.

**Table 4:** Pairwise Spearman correlations among fitness components

	Liquid Growth	Slug Distance	Total FB	Spores per FB	Total Spores	Spore Germination	Competitive Ability
Plate growth	−0.03	−0.13	0.22	−0.08	0.06	−0.07	0.00
Liquid growth		−0.06	−0.11	−0.04	−0.16	0.04	−0.09
Slug distance			0.17	−**0.40**	−0.26	0.11	−0.04
Total FB				−0.25	**0.37**	−0.02	−0.05
Spores per FB					**0.62**	−0.15	0.02
Total spores						−0.23	−0.08
Spore germination							0.08

Significant correlations at *P* = 0.05 level after we corrected for multiple comparisons (Benjamini and Hochberg 2000) are shown in bold. FB, fruiting body.

Our observed data, with possible trait dependencies, matched simulated data with independent trait effects, again suggesting that pleiotropy was absent or rare in our data. When we randomized the observed number of significantly different fitness components across lines, the resulting distribution was significantly different (*α* = 0.05) from the observed distribution in only 69 of the 1000 simulations. Thus the observed distribution of lines containing 0–8 significantly different fitness components is consistent with independence of fitness components in 93% of simulations. Likewise, when we estimated mutation rate for each component and then used those to simulate mutations for each fitness component, we found that 77 of the 1000 simulations were significantly different. The observed distribution of lines containing 0–8 significantly different fitness components is consistent with independence of fitness components in 92% of simulations. In Table 5, we show the number of lines possessing 0–8 significantly different fitness components both for the observed data and for the average of the simulated data when the numbers of fitness-altering mutations can vary.

**Table 5:** The observed and expected number of MA lines exhibiting 0–8 fitness values that are significantly different from the ancestor distributions

Affected Components	Observed MA Lines	Expected from Simulation
0	0	0.01
1	1	0.31
2	4	3.23
3	13	13.59
4	24	28.33
5	34	29.12
6	11	13.38
7	3	2.00
8	0	0.04

The expected values are derived by simulation as described in the text. There is no significant difference between the two distributions (χ^2^ = 4.26, *df* = 8, *P* = 0.83). MA, mutation accumulation.

We note that the sum of the mutation rates for each fitness component, estimated from the number of MA lines that are not significantly different from the ancestor, gives an average of 9.0 mutations per MA line. With nine mutations, we should perhaps expect to see all of the eight fitness components altered in all MA lines. However, multiple mutations can affect a single fitness component in some MA lines, so that not all components are affected.

### Parameters of mutation

Parameters of mutation estimated by ML and Bateman-Mukai methods are shown in Table 6. For two factors (plate growth and competitive ability), the Bateman-Mukai method could not be applied. In both cases the difference in variance between the MA line and ancestor distributions was greater than the difference in mean, which violates a requirement of the method. For spore germination, the likelihood surface was very flat over a broad range of values, probably because the ancestor shows essentially no variation for this measure (all spores germinate), whereas the MA lines exhibits a great deal of variation (Figure 1). After attempting multiple parameter combinations, the likelihood analysis of spore germination was abandoned.

**Table 6:** ML (columns 2-4) and BM (columns 5 and 6) estimates of mutation parameters for each fitness component

Component	P (ML)	E(*a*) (ML)	U (ML)	E(*a*) (BM)	U (BM)
Plate growth	0.3 (0.03–0.70)	0.112*^a^* (0.022–0.150)	0.0001 (0.00003–0.0007)	*^b^*	*^b^*
Liquid growth	0.15 (0.03–0.46)	0.11 (0–0.135)	0.0039 (0.0024–∞)	0.156	0.0019
Slug distance	0.3 (0.15–0.42)	0 (0–0.11)	∞ (0.0050–∞)	0.206	0.0005
Total FBs	0.4 (0.25–0.48)	0.055 (0–0.086)	0.0027 (0.0012–∞)	0.311	0.0002
Spores per FB	0.4 (0.25–0.42)	0 (0–0.12)	∞ (0.0019–∞)	0.338	0.0002
Total spores	0 (0–0.38)	0.052*^a^* (0–0.082)	0.0026 (0.0014–∞)	0.053	0.0025
Spore germination	*^c^*	*^c^*	*^c^*	0.148	0.0033
Competitive ability	0.3 (0.22–0.46)	0.23 (0.08–0.26)	0.0015 (0.0014–0.0031)	*^b^*	*^b^*

For ML estimates, the 2 log unit support intervals are shown in parentheses. BM estimates are only calculated for the six components in which the difference in the mean between the MA lines and ancestor distributions was greater than the difference in the variance. Effect can be positive or negative for ML estimate, and is negative (deleterious) for BM estimates. ML, maximum likelihood; BM, Bateman-Mukai; *P*, proportion beneficial; E(*a*), average fitness effect of a substitution; *U*, haploid genome-wide mutation rate per cell generation; MA, mutation accumulation.

a Equal effects gives highest likelihood.

b BM is not applicable.

c Data are poor fit to assumptions of likelihood.

For two components of fitness (slug distance and spores per FB), the likelihood continues to increase as the number of mutations increases and their average effect decreases. An estimate for these two parameters for these data sets thus cannot be given. This problem is a common issue in MA experiments (*e.g.*, Keightley and Ohnishi 1998). Similarly, the likelihood surface often flattens as the mutation rate estimate becomes large and the average effect decreases, which implies that distinguishing many mutations of tiny effect from fewer of larger effect is not possible. The result is that it is often the case that only one side of the confidence intervals is obtained for both the mutation rate (lower bound) and the average effect (upper bound). This was observed for all of the fitness components except growth in the experimental conditions (plate growth) and competitive fitness (competitive ability).

For six of the seven fitness components for which likelihood estimates could be obtained, there is striking agreement among the ML estimates and/or lower bounds for the genome-wide mutation rate to alleles altering fitness, *U*, with all falling within the range 1.2–5.0 × 10^−3^ mutations per generation. This corresponds to an average of 1.2–5.0 mutations affecting each fitness component that accumulated in each MA line. The exception is for growth in the experimental conditions (plate growth). The ML value for this component is 10-fold less than estimates for other components, and the upper bound is 2- to 3-fold less than the lower bound for the other components. The estimated mutation rate lower bounds can be used to estimate the total number of mutations arising in a line. This estimate is ~13 mutations per line and is reassuringly close to the 9.0 mutations per line obtained from the point estimates used in the simulations. There is also good agreement among the ML estimates and/or upper bounds for the average effects of a mutation affecting fitness, E(*a*), with all in the 0.05–0.26 range.

The Bateman-Mukai estimates were generally higher for effect size and lower for mutation rates, which is as expected if there is variation in the effect sizes (Lynch *et al.* 1999). The one exception is for total spores produced in which the Bateman-Mukai estimate is essentially identical to the ML estimate because the ML estimate yields the equal effects model, which is the underlying distribution of effects assumed in the Bateman-Mukai method.

One of the most surprising observations is the high estimate for the frequency of beneficial mutations observed across six of the fitness components (Table 6). Only one fitness component (total spores) has an estimate of no beneficial mutations (*P* = 0) within the confidence intervals of the ML estimate, although two others are close, with lower confidence intervals = 0.03 for both plate growth and liquid growth.

There are other ad hoc methods for estimating the mutation rate and the frequency of beneficial mutations. For the mutation rate, the fact that no MA Line exists that has all eight fitness components within the range of the ancestor implies that every MA line had at least one fitness-altering mutation. This allows a lower bound on the mutation rate to be calculated by determining the mutation rate (in a Poisson process) at which the probability of obtaining zero no-mutation lines in 90 MA lines is less than 5%. This analysis gives a lower estimate of *U* as 0.01 mutations per generation, or ~10 mutations accumulated per line. For the frequency of beneficials, one can simply calculate the percentage of MA lines that are significantly different from the ancestor (Table 3), which gives a range of estimates for *P* from 0 to 28% across the eight fitness components. Both of these estimates are consistent with those from the ML analysis.

## Discussion

### Mutation accumulation

MA experiments are designed to minimize the effects of selection. However, even at low effective population size (*N_e_* = 7.5), mutations with effects on the order of the reciprocal of the effective size will be selected. In this experiment, 1/ *N_e_* = 0.13, which is close to the mean effect size for the only trait that could be directly selected growth rate in the experimental condition (plate growth; mean effect size = 0.112; Table 6). This finding suggests that deleterious mutations with effect sizes greater than the mean would not have accumulated very efficiently. This result is consistent with the response of this trait, which showed no decrease in the mean or increase in the variance after MA (Table 2). For this reason, estimates of mutation parameters for this fitness component are likely biased downward for both mutation rate and effect size.

In the absence of pleiotropy, the other fitness components that were measured should not be affected by selection occurring in the MA condition. No other fitness component was directly selected during MA: cells were never grown in axenic medium (liquid growth), nor were they allowed to fruit (all other fitness components). Given the lack of evidence for extensive pleiotropy (see *Pleiotropy* below) selection apparently did not operate on these traits.

The control lines experienced much larger effective population size than the MA lines. If growth in the experimental condition (plate growth) was under selection, then there should be no differences in the control and the ancestor distributions because selection will prevent the accumulation of deleterious alleles, which is what was observed. For other components of fitness, which were not directly selected, we expect the control lines and the MA lines to be similar if there is no pleiotropy. We observed this for two fitness components (competitive ability and slug distance) but not for the remaining five components. The mean value of the control lines was intermediate between the MA lines and the ancestor for two components (total spores and spore germination) and not significantly different from the ancestor for another two (liquid growth and spores per FB). One possibility is that there is a class of rare, small-effect, positively pleiotropic mutations that are beneficial in the MA environment (plate growth). These mutations will be sampled in the control experiment, but not in the MA lines, because the effective population size is larger in the control lines. Beneficial mutations will increase in frequency in the control lines, and if they are pleiotropic, their presence will offset fitness reductions across other fitness components, causing control lines to show less fitness decay. For one fitness component, the total number of FBs, the mean of the control lines is significantly lower than both the ancestor and MA lines (Table 2 and Figure 1). It is not clear to us why this was the case.

The total number of FB also showed no difference in mean between the ancestor and MA lines distributions. One explanation for this observation is that this fitness component is under stabilizing selection in nature; therefore, new mutations cause the trait to change in either direction. If this explanation is true, among-line variance should be greater than among-replicate variance in the ancestor, which is the case (Table 2). This possibility is considered again below in *Proportion beneficial*.

### Parameters of mutation

#### Mutation rate (U):

Using the lower bounds of the mutation rates for each fitness parameter, we can place a lower bound on the overall rate of mutations that alter some component of fitness in *D. discoideum* by summing them. The sum of the lower bounds from the ML analysis is 0.0133 mutations per haploid genome per generation, which corresponds to an average of 13.3 mutations accumulated per MA line. This is based on seven of the eight fitness components. If the mutation rate estimate for spore germination is similar to the other components, then the estimate should be bumped up to 0.0152 mutations per haploid genome per generation, corresponding to 15.1 mutations accumulated per MA line. This estimate is reassuringly similar to the estimate of 9.0 mutations obtained from the point estimates used in the simulations.

For six of the seven fitness components for which there are ML estimates, the average lower bound of the mutation rate is 2.2 × 10^−3^ mutations per haploid genome per generation. For growth in the experimental medium (plate growth), the value is substantially lower, 3 × 10^−5^, presumably because of selection (see *Mutation accumulation* above). These values are an order of magnitude greater than the estimates for various fitness components in *S. cerevisiae* (Table 1). Instead, they are more similar to mutation rate estimates in nematodes (Table 1). The high rate of fitness-altering mutations suggests that the molecular mutation rate is higher in *D. discoideum* and/or a larger percentage of molecular mutations alters fitness.

There are data addressing the molecular mutation rate. Nucleotide diversity (*π*) is expected to equal 4 *N*_e_*μ* at equilibrium, where *N*_e_ is the effective population size and *μ* is the base-pair substitution rate (Hartl and Clark 2007). Estimates of *π* in *D. discoideum* vary from a low of 0.0008 (Flowers *et al.* 2010) to a high of 0.04995 (Francis and Eisenberg 1993) and partly overlap the range for *S. cerevisiae*, which is from 0.008 (Fay *et al.* 2004) to 0.068 (calculated in Lynch 2006, using data from Sakai *et al.* 1992). However, the smallest estimate for *D. discoideum* is based on the most comprehensive data set and suggests that *π* is perhaps an order of magnitude smaller than in *S. cerevisiae*. Given this estimate, the base-pair substitution rate in *D. discoideum* can only be larger than in *S. cerevisiae* if its effective population size is more than 10-fold smaller. A direct estimate of the base-pair mutation rate (Saxer *et al.* 2012), obtained by sequencing three of the MA lines discussed here, was very low (2.9 × 10^−11^ per base). This finding suggests that the molecular basis of the high rate of fitness-altering mutations does not lie in base-pair substitutions.

The mutational target size to alleles that alter fitness in *D. discoideum* is larger than in yeast. *D. discoideum* has both more (9000 *vs.* 6200) and larger (2.45 *vs.* 1.44 kb) genes than *S. cerevisiae* (Lynch 2006). If the same percentage of mutations results in a fitness effect in the two species, then *U* should be approximately 2.5-fold larger in *D. discoideum* than *S. cerevisiae* based on mutational target size alone. This increase in coding region may be related to the increase in complexity (Haygood 2006) resulting from the presence of a multicellular life cycle stage.

In addition, *D. discoideum* has the highest density of microsatellite repeats of any sequenced organism, >11% of bases (Eichinger *et al.* 2005). Many of these are nonrandomly distributed among coding regions: for example tracts of 20 or more asparagine or glutamine residues are present in 2,091 of the predicted proteins (Eichinger *et al.* 2005). In humans, triplet repeats near or inside of coding regions are sometimes subject to expansions, which can directly cause genetic diseases (Cummings and Zoghbi 2000; Warren and Ashley 1995). It is unknown whether *D. discoideum* suffers deleterious effects from these exonic coding regions, but previous work suggests that these microsatellites are highly variable (Scala 2005; Scala *et al.* 2012). It is possible that the relaxation of selection during MA allows accumulation of mutations in microsatellites that have fitness effects. The microsatellite mutation rate for this species, 6.37 × 10^−6^ per generation (McConnell *et al.* 2007), is the lowest ever reported, indicating that it is not mutation rate *per se* but the large number of mutational targets that might be responsible for the elevated *U*.

An important question is whether the observed mutation rate is shared by other protists, or whether it is unique to *D. discoideum*. Data are available for only a couple of species. One MA study performed in a sexual species of ciliate, *Tetrahymena thermophila*, found rapid loss in fitness, and extinction of all 20 MA lines after only 16 transfers (Brito *et al.* 2010). The rapid fitness loss was hypothesized to be due to mutational decay caused by chromosomal copy number changes in the macronucleus. The chromosomal loss/gain rate was estimated to be ~0.033 per haploid genome, which is similar in magnitude to our estimate. However, there is no estimate for the micronucleus, which would be more appropriate to compare to the values we found in *D. discoideum*, which lacks a macronucleus. A MA study in *Paramecium tetraurelia* gave a base pair substitution rate estimate of ~1.9 × 10^−11^ per cell division (Sung *et al.* 2012), which is similar to *D. discoideum*. It would be interesting to determine the genome-wide rate of fitness-altering mutations (*U*) in this species. The prediction is that if microsatellites are rare, *U* will be low, reflecting the base-pair substitution rate.

#### Average effect [E(a)]:

The estimated effect sizes for *D. discoideum* differ across the fitness components, although there is substantial overlap, with some toward the higher end of previous estimates (Table 1, competitive ability). One explanation for large effect estimates is that some fitness components were not directly selected because they were not induced during MA. Support for this conclusion comes from the observation that the largest effect size is for the competitive fitness component (competitive ability). In addition, only two MA lines were significantly different from the ancestor for growth in the experimental medium (plate growth; Table 3), where selection would have been more effective in reducing the accumulation of large-effect deleterious mutations.

#### Proportion beneficial (P):

Although most mutations were deleterious, there was evidence in six fitness components of mutations with beneficial (increased) fitness effects as indicated by higher fitness MA lines. Estimates of the proportion of mutations that were beneficial ranged from 0 to 40% from the ML analysis (Table 6) and from 0 to 28% from MA lines that were significantly different from the ancestor (Table 3). Does this observation imply that a large percentage of mutations in nature are advantageous? There are three reasons that make such a conclusion tentative. First is the observation that the fitness components that are arguably the closest to capturing total fitness, growth rate, both on bacteria and in axenic medium, and spore number, have the least evidence for a high frequency of beneficial mutations. The ML estimate for the proportion beneficial for total spores is zero, and the lower bounds of the ML estimates are very near zero for growth on bacteria and growth in axenic medium (Table 6). In addition only a single line for growth in axenic medium and for spore number, and no lines for growth on bacteria had significantly higher fitness than the ancestor (Table 3). These data suggest that these fitness components have been directionally selected for high values in the past in nature, or in the laboratory, and accumulated mutations thus reduce the values of these components. If these are the primary fitness components acted on by selection, then the conclusion is that accumulated mutations were almost exclusively deleterious.

Second, some traits may be under stabilizing selection in nature because of their effects on spore production. The number of FBs and the number of spores are positively correlated (Table 4). However, if the relationship between spore number (*i.e.*, fitness) and FB has a nonlinear component, that could indicate stabilizing selection. In that situation, we would expect significant nonlinearity, with an optimum close to the ancestral value. Put another way, mutations that increase or decrease the number of FBs relative to the ancestor may all decrease spore number. To examine this hypothesis, a nonlinear regression between spore number and FB number was calculated by fitting a cubic relationship. This analysis indicated a significant nonlinearity, with a local optimum occurring at a relative fitness of 1.06 (Figure S2A). A similar nonlinear regression analysis was also performed for spores per FB, which is also positively correlated with spore number (Table 4). Again there was significant nonlinearity, with a maximum at 1.21 (Figure S2B). These results suggest that both of these fitness components may be under stabilizing selection, which would imply that mutations that sufficiently increase the value of one of these fitness components are not in fact beneficial overall. None of the others fitness components exhibited significant nonlinearity in a regression with spore number. This supports the idea that it is stabilizing selection on spores per FB, and total number of FBs, that causes the high frequency of “beneficial” mutations that increase these trait values.

Third, the high frequency of trait-increasing mutations may indicate the trait is not actually a fitness component. If correct, the ancestor’s particular trait value has little predictive power regarding the effects of mutations. This is because there has been no evolutionary history of selection removing only those mutations that reduce the trait value, as there has for directionally selected fitness traits, which enriches them among random mutations. For the remaining two components exhibiting high estimates of the frequency of trait-increasing mutations, the distance that a slug travels and the relative competitive ability, is this a likely explanation? Slug migration is thought to be an important component of fitness in *D. discoideum* (Foster *et al.* 2002). Slug migration allows *D. discoideum* to move to regions of more abundant resources (Kuzdzal-Fick *et al.* 2007). It also assists in long-range spore dispersal and may assist in predator protection (huss 1989f1; Kessin 2001; Kessin *et al.* 1996). The fact that the sizeable majority of mutations reduced slug distance supports the notion that slug migration distance being at least partially under direct selection in nature.

Competitive ability is a particularly interesting trait in *D. discoideum*. Unlike most multicellular organisms, a *Dictyostelium* FB can be composed of multiple clones, and it has emerged as a model system of conflict and cooperation (Strassmann and Queller 2011b). Cheating is clearly important in the lab (Ennis *et al.* 2000; Khare *et al.* 2009; Kuzdzal-Fick *et al.* 2011; Santorelli *et al.* 2008) and there is some evidence of apparent conflict adaptations – for example social hierarchies (Buttery *et al.* 2009; Fortunato *et al.* 2003a), costs of chimerism (Castillo *et al.* 2011; Foster *et al.* 2002), competition strategies (Kuzdzal-Fick *et al.* 2011), and kin discrimination (Benabentos *et al.* 2009; Ostrowski *et al.* 2008). However, our results suggest that competitive ability is a rather weak fitness component in nature. It does decline under MA, as expected if it is a fitness component, but it also shows the highest number of lines with beneficial mutations of any trait. The likely explanation seems to be that competitive ability is a minor fitness component with a history of relatively weak directional selection. This result is consistent with the finding that most FBs in nature are clonal and only a minority are mixed (Gilbert *et al.* 2007). Selection on competitive ability would therefore be relatively rare and consequently relatively weak (Van Dyken and Wade 2010). Compared with other traits, fewer of the possible beneficial mutations would already have been fixed.

We suggest that this logic might be applied to other cases. It is often difficult to tell what traits are fitness components, particularly for microbes, which are difficult to study in a natural environment. The asymmetrical response of fitness-related traits under MA can provide a novel line of evidence on this score.

In summary, for the six fitness components showing evidence for non-zero frequencies of beneficial mutations, *i.e.*, those that increase the value of the fitness component, two show low frequencies of these mutations (affecting just a single MA line), two are consistent with stabilizing selection, implying the increased value for the component is likely deleterious, and just two (slug distance and competitive ability) have a high frequency of *bona fide* beneficial mutations, one of which is likely under weak selection in nature. The frequency of truly beneficial mutations, *i.e.*, those that would be favored in nature, may thus be lower in *D. discoideum* than a cursory examination of Table 3 or Table 6 would suggest.

### Pleiotropy

There are three reasons to conclude that accumulated mutations exhibit little pleiotropy across fitness components: there are few significant correlations between fitness components (Table 4); the effective number of traits is very close to the actual number measured; and the distribution of multiply affected lines is consistent with independence among mutations (Table 5). This evidence strongly suggests that MA lines that are significantly different from the ancestor for multiple fitness components carry multiple, independently acting mutations. This finding of low pleiotropy is consistent with previous MA studies in which investigators have looked for evidence of pleiotropy (Hall and Joseph 2010) and for other studies using knockouts or other mutations to categorize levels of pleiotropy (reviewed in Wagner and Zhang 2011). However, it is perhaps surprising that there are no mutations that cause general unfitness, which would reduce the fitness of all components. One reason that such a class of mutation seems to be missing may simply be that the effect size for growth in the experimental condition (plate growth) is large for this class of mutations. If this were the case, then this class of generally unfit mutations would not have been able to accumulate during MA because of selection, which would explain their absence.

In summary, we accumulated mutations in 90 *D. discoideum* lines for 994 generations under conditions that minimized natural selection. We measured the change in eight fitness components for each MA line and estimated that the rate of mutation per haploid genome per generation affecting a fitness component is ~0.0019 or larger. This estimate is an order of magnitude higher than in *S. cerevisiae*, even though *D. discoideum* has a base pair substitution rate that is two orders of magnitude lower; 2.9 × 10^−11^ mutations per generation (Saxer *et al.* 2012), equivalent to 0.001 mutations per haploid genome per generation. *D. discoideum* has the highest density of microsatellite repeats in any sequenced genome, ~11% of bases, and more than 2000 protein coding genes possess repeats of 20 or more amino acids, which suggests that it is mutations at microsatellite repeats that are the likely basis of the fitness-altering mutations.

## Supplementary Material

Supporting Information
