# Supplementary material for: The Rate and Effects of Spontaneous Mutation on Fitness Traits in the Social Amoeba, Dictyostelium discoideum
Source: G3 (Bethesda). 2013 Jul 1;3(7):1115–27. doi: 10.1534/g3.113.005934 (PMC3704240; doi:10.1534/g3.113.005934)
Supplement: Supporting Information [file supp_3_7_1115__index.html]

The Rate and Effects of Spontaneous Mutation on Fitness Traits in the Social Amoeba, Dictyostelium discoideum — Supporting Information 

# The Rate and Effects of Spontaneous Mutation on Fitness Traits in the Social Amoeba, *Dictyostelium discoideum*

## Supporting Information for Hall *et al.*, 2013

**Files in this Data Supplement:**

- Supporting Information - Figures S1 and S2 (PDF, 305 KB)
- Figure S1 - Distributions of ancestors and MA lines for eight putative fitness components (PDF, 191 KB)
- Figure S2 - (A) Cubic regression of spore number on number of fruiting bodies (B) Cubic regression of relative total spore number on spores per fruiting body (PDF, 104 KB)
