# Supplementary material for: The Rate and Effects of Spontaneous Mutation on Fitness Traits in the Social Amoeba, Dictyostelium discoideum
Source: G3 (Bethesda). 2013 Jul 1;3(7):1115–27. doi: 10.1534/g3.113.005934 (PMC3704240; doi:10.1534/g3.113.005934)
Supplement: Supporting Information [file supp_g3.113.005934_005934SI.pdf]

G3 · Genes | Genomes | Genetics

The rate and effects of spontaneous mutation on fitness traits in the social amoeba, *Dictyostelium discoideum*

David W. Hall*, Sara Fox§, Joan E. Strassmann†, and David C. Queller†.

* Department of Genetics, University of Georgia
§ Department of Ecology and Evolutionary Biology, Rice University
† Department of Biology, Washington University in St. Louis

DOI: 10.1534/g3.113.005934

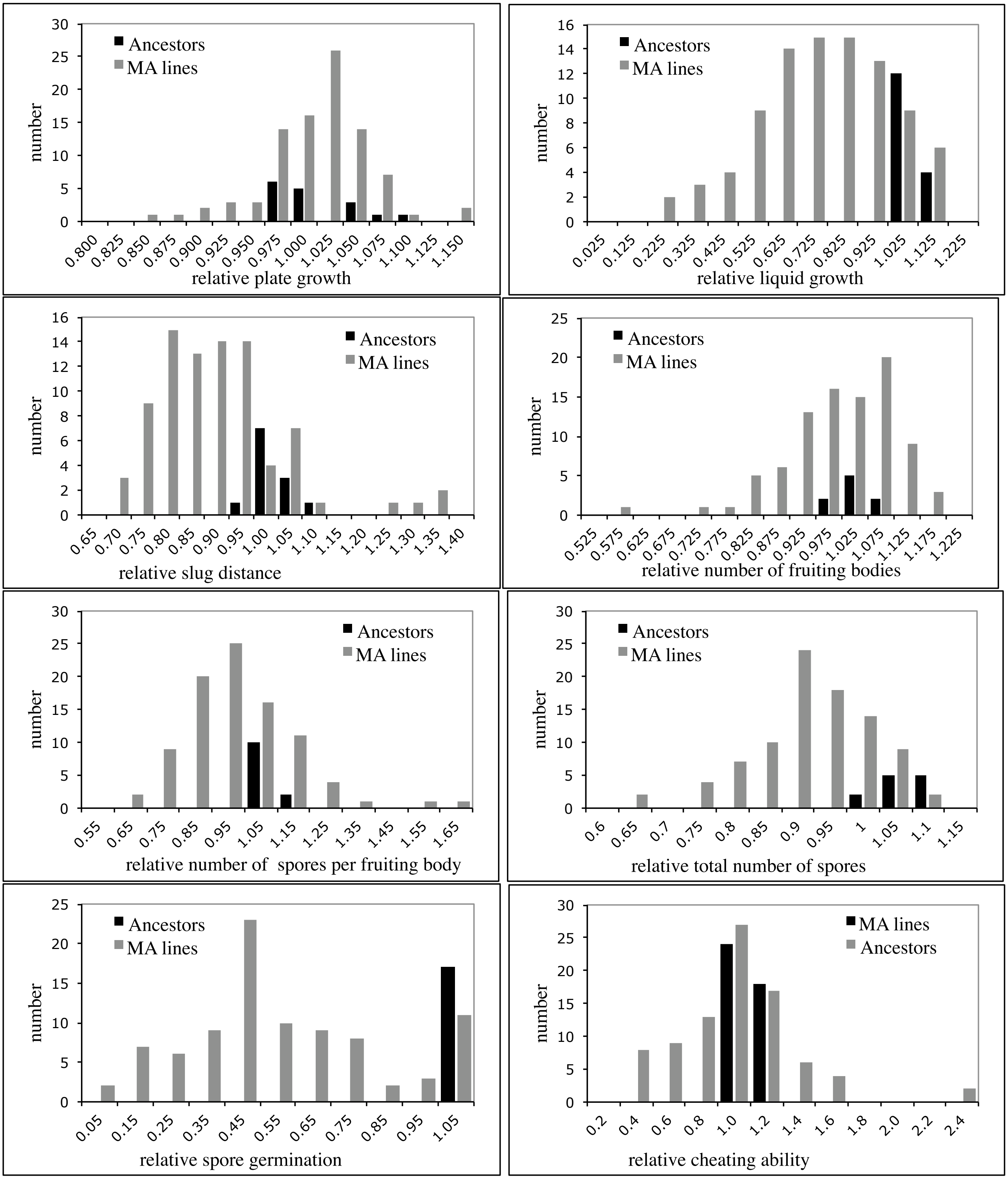

Figure S1 Distributions of ancestors and MA lines for eight putative fitness components.

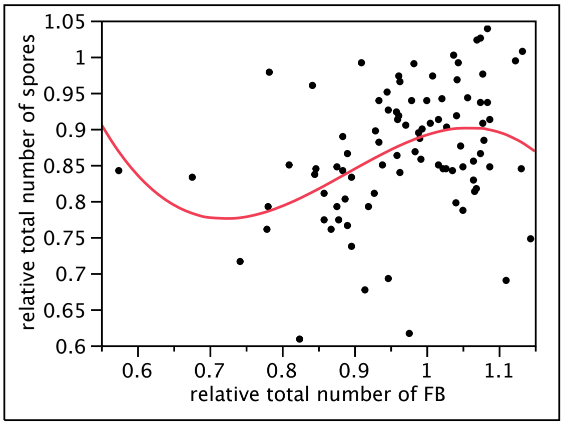

Term	Estimate	Std Err	t Ratio	Prob>|t|
Intercept	0.453	0.115	3.95	0.0002*
Relative total # of fruiting bodies	0.440	0.119	3.70	0.0004*
(Relative total # of fruiting bodies - 0.96858)^2	-1.546	1.003	-1.54	0.1270
(Relative total # of fruiting bodies - 0.96858)^3	-6.579	3.220	-2.04	0.0441*

Figure S2A Cubic regression of spore number on number of fruiting bodies. Significant cubic term indicates non-linearity and is consistent with stabilizing selection on number of fruiting bodies.

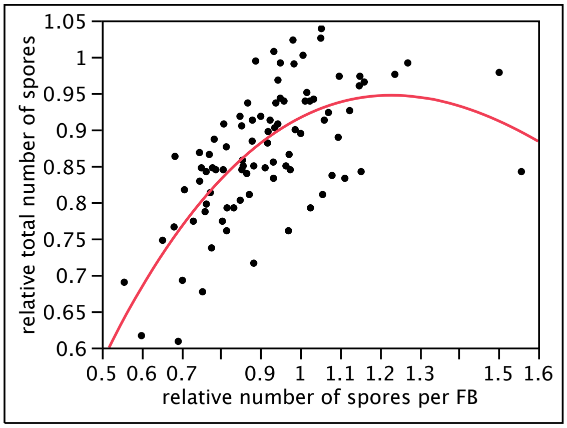

Term	Estimate	Std Err	t Ratio	Prob>|t|
Intercept	0.527	0.059	9.00	<.0001*
Relative # spores per fruiting body	0.393	0.062	6.34	<.0001*
(Relative # spores per fruiting body - 0.91935)^2	-0.728	0.238	-3.06	0.0030*
(Relative # spores per fruiting body - 0.91935)^3	0.205	0.494	0.41	0.6797

Figure S2B Cubic regression of relative total spore number on spores per fruiting body. Significant quadratic term indicates non-linearity and is consistent with stabilizing selection on relative number of spores per fruiting body.
