# Supplementary figures and images for: The Rate and Effects of Spontaneous Mutation on Fitness Traits in the Social Amoeba, Dictyostelium discoideum
Source: G3 (Bethesda). 2013 Jul 1;3(7):1115–27. doi: 10.1534/g3.113.005934 (PMC3704240; doi:10.1534/g3.113.005934)

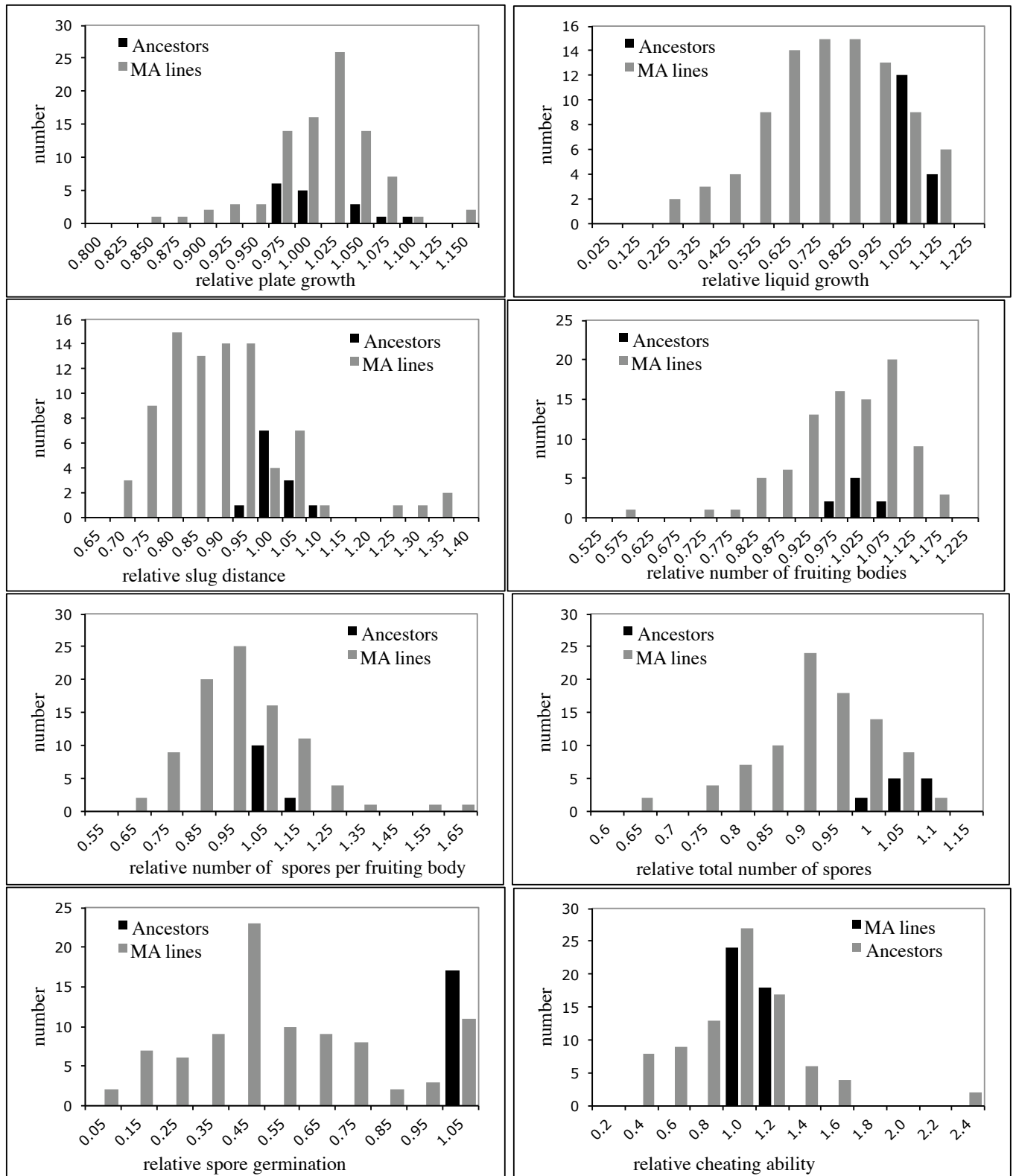

**Figure S1** Distributions of ancestors and MA lines for eight putative fitness components.

Supplement: Supporting Information [file supp_g3.113.005934_FigureS1.pdf]
